# Pathophysiology and Comprehensive Pharmacological Management of ATTR Cardiac Amyloidosis: Knowledge Gaps, Ongoing Clinical Trials, and Future Research Directions

**DOI:** 10.3390/ph19101542

**Published:** 2026-09-29

**Authors:** Sareen Bains, Harry Atwal, Ashbir Sidhu, Harpreet Singh, Gurpreet Sodhi

**Affiliations:** 1 School of Medicine, St. George’s University, St. George’s, Grenada; asidhu5@sgu.edu; 2 Cardiovascular Disease, Sutter Roseville Medical Center, Roseville, CA 95661, USA; harpreet.singh4@sutterhealth.org; 3 Advanced Heart Failure and Transplantation, Sutter Sacramento Medical Center, Sacramento, CA 95816, USA; gurpreet.sodhi@sutterhealth.org; 4 School of Medicine, California Northstate University, Elk Grove, CA 95757, USA

**Keywords:** transthyretin cardiac amyloidosis, ATTR-CA, TTR stabilizers, Tafamidis, Acoramidis, gene silencing, Vutrisiran, Eplontersen

## Abstract

The purpose of this review article is to discuss the current treatment modalities and ongoing clinical trials regarding cardiac amyloidosis (CA), specifically transthyretin cardiac amyloidosis (ATTR-CA). Amyloidosis refers to the extracellular deposition of insoluble amyloid fibrillar proteins. When these proteins are deposited in the heart’s myocardium, it is referred to as cardiac amyloidosis. CA can be divided into two main groups: light chain cardiac amyloidosis (AL-CA) and transthyretin cardiac amyloidosis (ATTR-CA), which can be further subdivided into wild-type transthyretin amyloidosis (ATTRwt) and hereditary transthyretin amyloidosis (ATTRv). ATTR-CA is an increasingly recognized cause of heart failure in the setting of progressive restrictive cardiomyopathy, particularly among older adults, males, and individuals of African descent, with remarkable advances in the therapeutic landscape. TTR stabilizers, including Tafamidis (approved in 2019) and Acoramidis (approved in 2024), have demonstrated significant reductions in mortality and cardiovascular hospitalizations. Gene silencing therapies, including Vutrisiran (approved in 2025), represent a new drug group that targets TTR production at the RNA level. Meanwhile, CRISPR-based gene editing with nexiguran ziclumeran (nex-z or NTLA-2001) and amyloid-depleting monoclonal antibodies offer the prospect of one-time curative treatments and active fibril clearance, respectively. However, despite these advances, substantial knowledge gaps persist: no head-to-head trials have compared stabilizers to silencers, optimal combination therapy regimens remain undefined, and the role of conventional guideline-directed medical therapy in ATTR-CA has only been supported by observational data.

## 1. Introduction

Systemic amyloidosis is a group of progressive disorders characterized by the extracellular deposition of amyloid fibrils derived from unstable proteins that misfold and aggregate into insoluble β-sheets [1]. A total of 42 precursor proteins has been identified as capable of forming amyloid fibrils, serving as the basis for their classification [2]. Their clinical manifestations vary according to the amyloid type, the burden of deposition, and the tissues involved. When the heart is the target of this infiltrative process, it is termed cardiac amyloidosis (CA) [3,4]. CA is caused predominantly by either plasma cell dyscrasias producing misfolded immunoglobulin light chains (AL-CA) or by transthyretin (TTR) amyloidosis (ATTR-CA), which may arise from age-related or hereditary causes [5]. Both forms may result in a restrictive cardiomyopathy due to amyloid fibril deposition within the myocardium, leading to progressive diastolic and systolic dysfunction, conduction abnormalities, and heart failure [6].

TTR is synthesized predominantly by the liver and functions as a transport protein for thyroxine and the retinol–retinol-binding protein complex 4 [7]. In its wild-type form (ATTRwt), formerly known as senile cardiac amyloidosis, disease results from an incompletely understood, age-related dissociation of the TTR tetramer and predominantly affects older adults, with a mean age at diagnosis of 74–78 years in men [8]. Hereditary transthyretin amyloidosis (ATTRv) is caused by autosomal dominant, typically missense mutations in the TTR gene that destabilize the tetramer and promote its dissociation into partially unfolded monomers that aggregate into amyloid fibrils [9]. More than 150 pathogenic TTR variants have been identified [10]; among these, the Val122Ile mutation is the most common variant in the United States, occurring in ~3.4% of African Americans and predominantly associated with a cardiac phenotype [11]. In contrast, the Val30Met mutation is the most prevalent variant worldwide, with large endemic foci in Portugal, Sweden, and Japan [12]. This genetic heterogeneity contributes to substantial variability in age of onset, organ involvement, and disease severity among affected individuals [12].

### Structural Biology and Pathophysiology of Transthyretin

Mature human transthyretin (TTR), formerly called prealbumin, is a 127-amino-acid protein with a monomeric molecular mass of approximately 13.8 kDa. Under physiologic conditions, four identical subunits assemble into a soluble homotetramer of approximately 55 kDa. Each monomer contains eight antiparallel beta strands, designated A through H, arranged as two four-stranded beta sheets (DAGH and CBEF) that form a beta-sandwich, together with a short alpha helix. Two monomers associate through an extensive hydrogen-bond network to form a dimer, and two dimers pack across the comparatively weaker dimer-dimer interface to form the functional tetramer and its two central thyroxine-binding channels. This quaternary architecture permits transport of thyroxine and the retinol–retinol-binding protein complex [7,8]. The hepatic biosynthesis, post-transcriptional processing, translation, folding, and tetrameric assembly of TTR are summarized in Figure 1 [7,8].

Amyloidogenic vulnerability is associated with residues and structural regions that maintain the hydrophobic core, beta-sheet packing, flexible loops, and monomer-monomer and dimer-dimer interfaces. Pathogenic substitutions occur throughout the TTR sequence, but Val30Met and Val122Ile are among the most clinically important variants. These single-residue substitutions reduce conformational stability and influence tissue tropism: Val30Met is commonly associated with peripheral and autonomic neuropathy, whereas Val122Ile predominantly produces a late-onset cardiac phenotype [9,10,11,12]. Variant position alone does not determine disease expression because age, genetic background, proteostasis, and local tissue conditions also influence penetrance, age at onset, and organ involvement. Wild-type TTR may undergo similar age-related destabilization in the absence of a pathogenic coding variant [7,8,9,10].

TTR fibrillogenesis begins with dissociation of the native tetramer, which is considered the rate-limiting step in amyloid formation. Released monomers partially unfold and undergo beta-sheet rearrangement, exposing aggregation-prone surfaces that assemble into soluble oligomers, protofilaments, and ultimately insoluble cross-beta amyloid fibrils. Both full-length and proteolytically fragmented TTR may be incorporated into cardiac deposits. Mature fibrils can also fragment and seed further fibril growth [7,8,13].

In the heart, extracellular fibril accumulation expands the myocardial interstitium and disrupts tissue architecture, causing increased ventricular wall thickness, chamber stiffness, restrictive filling, declining stroke volume, conduction disease, arrhythmias, and progressive heart failure. Soluble prefibrillar species may additionally exert direct cellular toxicity, while vascular deposition may contribute to microvascular dysfunction. Because ATTR is systemic, deposition outside the heart can produce peripheral and autonomic neuropathy, carpal tunnel syndrome, lumbar spinal stenosis, and biceps tendon rupture [5,7,8,9,10].

Although ATTR-CA was historically considered a rare and difficult-to-diagnose disease, advances in noninvasive imaging and growing clinical awareness have led to increased recognition of transthyretin amyloid cardiomyopathy (ATTR-CM) as an underdiagnosed cause of heart failure (HF) [5]. In a retrospective cohort from the Veterans Health Administration of 2557 patients diagnosed with both ATTR-CM and HF between 2016 and 2022, the median diagnostic delay between initial HF diagnosis and ATTR-CM diagnosis was 490 days, with more than 25% of patients experiencing delays exceeding 3 years. Atrial fibrillation, coronary artery disease, and chronic kidney disease were associated with increased odds of delayed diagnosis [13]. A separate targeted literature review reported misdiagnosis rates of 34–57% and weighted mean diagnostic delays of 6.1 years for ATTRwt and 5.7 years for ATTRv [14]. These delays, attributed to nonspecific presentation, overlap with common age-related cardiac conditions, and limited clinician awareness, postpone access to disease-modifying therapies, which are most effective when initiated prior to significant cardiac dysfunction [5,14].

Historically, ATTR-CM management was largely supportive, centered on symptomatic volume control and decongesting the patient [15]. However, because ATTR-CM is associated with restrictive left-ventricular filling and reduced stroke volume, aggressive diuresis risks acute kidney dysfunction in the setting of poor systemic perfusion. Conventional heart failure guideline-directed medical therapy (GDMT) is often poorly tolerated and has not been shown to modify the underlying process of TTR destabilization and amyloid deposition [5,16]. The development of therapies targeting the amyloidogenic cascade has shifted management from supportive care toward targeted intervention. In 2019, Tafamidis, a TTR tetramer stabilizer, became the first FDA-approved disease-modifying pharmaceutical for ATTR-CM following the landmark ATTR-ACT trial [17]. This was followed by the approval of Acoramidis, a second-generation stabilizer, in November 2024 [18], and by Vutrisiran in 2025, the first RNA interference–based gene-silencing therapy approved for ATTR-CM [19].

The therapeutic pipeline continues to expand, with several new approaches in various stages of clinical development. Investigational strategies include CRISPR-Cas9 gene editing, which offers the prospect of a single-dose, permanent reduction in TTR production [20]; monoclonal antibodies designed to clear existing myocardial amyloid; and advanced gene-silencing agents [15,21]. Despite these advances, significant knowledge gaps remain, including the lack of comparative studies among disease-modifying therapies, the undefined role and efficacy of combination stabilizer–silencer therapy, and uncertainty regarding the application of traditional GDMT alongside these new therapies [21]. This review focuses on the current and emerging pharmacological management of ATTR-CA, discussing their pharmacologic characteristics, clinical efficacy, and safety profile.

## 2. TTR Stabilizers

The thyroxine (T4)-binding channels at the dimer–dimer interface of the TTR tetramer are particularly important for conformational stability. Notably, fewer than 5% of circulating TTR molecules bind T4 under physiological conditions, leaving these channels largely unoccupied and available for pharmacological intervention [22]. When occupied by small-molecule stabilizers such as Tafamidis, the kinetic barrier to tetramer is raised. This prevents the rate-limiting step in amyloid formation [23]. By stabilizing the native quaternary structure, these agents slow or halt dissociation of the tetramer into amyloidogenic monomers without lowering circulating TTR concentrations.

### 2.1. Tafamidis

Tafamidis (Vyndaqel/Vyndamax) was the first FDA-approved disease-modifying, TTR-stabilizing therapy for ATTR-CM, approved in May 2019 [17]. Two bioequivalent formulations are available: Tafamidis meglumine 80 mg (4 × 20 mg capsules) once daily (Vyndaqel) and Tafamidis free acid 61 mg once daily (Vyndamax). Vyndamax is more readily available and the preferable formulation. The FDA-approved indication is to reduce cardiovascular mortality and cardiovascular-related hospitalization in patients with NYHA Class I-III symptoms caused by either ATTRwt or ATTRv; its efficacy in NYHA Class IV heart failure has not been established [16].

Tafamidis reaches peak plasma concentrations within approximately 4 h following oral administration and is not significantly affected by food intake. It has an average elimination half-life of 49 h, consistent with its once-daily regimen, and accumulates roughly 2.5-fold at steady state [24]. Tafamidis is more than 99% protein-bound, primarily to TTR itself, and is largely eliminated in the feces as the unchanged drug, with a smaller renal contribution as a glucuronide metabolite [24]. No clinically significant pharmacokinetic differences have been identified based on age, race/ethnicity, or personal degree of renal impairment. Moderate hepatic impairment reduces systemic exposure by approximately 40%, but tetramer stabilization is preserved because circulating TTR concentrations are proportionally lower in these patients. The effect of severe hepatic impairment remains unknown [24,25].

The pivotal Transthyretin Amyloidosis Cardiomyopathy Clinical Trial (ATTR-ACT) was a phase 3, multicenter, double-blind, placebo-controlled study that randomized 441 patients with wild-type and hereditary ATTR-CM in a 2:1:2 ratio to Tafamidis 80 mg, Tafamidis 20 mg, or placebo for 30 months [17]. Using the Finkelstein–Schoenfeld hierarchical method, Tafamidis demonstrated superiority over placebo in both co-primary endpoints: all-cause mortality (29.5% vs. 42.9%; hazard ratio [HR] 0.70; 95% confidence interval [CI], 0.51 to 0.96) and frequency of cardiovascular-related hospitalizations (relative risk ratio 0.68; 0.48 vs. 0.70 per year; 95% CI, 0.56 to 0.81), with a win ratio of 1.70 (95% CI, 1.26–2.29; *p* = 0.0006) [17,22]. This translated into a number needed to treat of approximately 7.5 to prevent one death over 30 months [8]. Patients receiving Tafamidis also experienced less functional decline, as measured by 6 min walk distance (a decline of 57 m vs. 133 m over 30 months), and preserved quality of life, determined by the Kansas City Cardiomyopathy Questionnaire Overall Summary (KCCQ-OS) score (a 21-point vs. 7-point decline) [8]. Efficacy was greater in participants with earlier-stage disease (NYHA class I–II), and survival curves began to separate after approximately 18 months of treatment [8,16].

Long-term follow-up has reinforced these findings. A long-term extension (LTE) study following ATTR-ACT studied patients that continued Tafamidis at the approved dose for an additional 60 months, compared to previous placebo patients that were switched to Tafamidis, who were termed the delayed treatment group [26]. At a median follow-up of 58.5 months, continuous Tafamidis (80 mg) was associated with significantly lower all-cause mortality than the delayed treatment group (44.9% vs. 62.7%; HR 0.59; 95% CI, 0.44–0.79; *p* < 0.001) [27], a benefit consistent across ATTRwt (HR 0.61; *p* = 0.006) and ATTRv (HR 0.57; *p* = 0.05) and observed in NYHA class III (HR 0.65; *p* = 0.06), though more robust in class I–II (HR 0.56; *p* = 0.003). A dose-comparison analysis also demonstrated a 30% relative reduction in the risk of death with 80 mg compared with 20 mg, establishing 80 mg as the optimal dose [26,28]. Post hoc echocardiographic analyses showed Tafamidis 80 mg was associated with a less pronounced decline in LV stroke volume, LV global longitudinal strain, and LV filling pressure over 30 months [29]. Because Tafamidis prevents, but does not reverse, amyloid deposition, it is expected to have greater benefit when administered early. Therefore, patients with limited survival expectancy are not ideal candidates [16].

In ATTR-ACT, Tafamidis was associated with a favorable side-effect profile, with adverse events reported as mild to moderate, and similar between the intervention and placebo groups regarding types of reaction and incidence [17]. Permanent discontinuation due to adverse events was less common in the Tafamidis groups than in the placebo group (0.8% vs. 2.3%) [8]. The most reported adverse effects include diarrhea and constipation, which were less frequent with Tafamidis than with placebo [8]. In real-world practice, routine safety monitoring has not been required [30]. However, an important drug interaction has been observed with statins: Tafamidis inhibits breast cancer resistance protein (BCRP), potentially increasing exposure to substrates such as Rosuvastatin. The FDA advises against concomitant Rosuvastatin (Crestor) use. If unavoidable, Rosuvastatin should be initiated at 5 mg, without exceeding 10 mg daily, due to the risk of rhabdomyolysis [24]. A retrospective study of 103 patients at Columbia University found that 12% of patients required a change in their statin regimen due to myalgias or elevated liver enzymes, with one case of rhabdomyolysis reported with Atorvastatin 80 mg [31].

The primary limitation of Tafamidis is its cost, with an annual list price of ~$225,000 in the United States, making it the most expensive cardiovascular drug at the time of its approval [32]. The 2022 AHA/ACC/HFSA heart failure guidelines had noted that Tafamidis provided “low economic value” at 2020 list prices [16]. For Medicare patients, annual out-of-pocket costs can approach $18,000 [5,30]. Commercially insured patients may access manufacturer copayment assistance programs; however, these are unavailable to Medicare or Medicaid beneficiaries due to anti-kickback statutes [30]. Independent charity assistance foundations exist but have income limits, leaving many patients unable to afford treatment [5].

### 2.2. Acoramidis

Acoramidis (Attruby) is the newest TTR stabilizer approved by the FDA in November 2024. Its treatment indications are to reduce cardiovascular death and cardiovascular-related hospitalization for ATTR-CM patients with either ATTRwt or ATTRv [18]. The FDA-approved dosage is 712 mg orally twice daily, administered as two 356 mg tablets per dose [33].

Acoramidis is rapidly absorbed, reaching peak plasma concentrations within approximately 1 h, and has a short effective half-life of ~6 h, therefore necessitating twice daily dosing. It is 96% protein-bound, primarily to TTR, and uses glucuronidation as the primary clearance pathway. Elimination occurs predominantly via urine and minimally through feces as its metabolites [33]. No dose adjustment is required for age, sex, race/ethnicity, or renal impairment, whereas the effect of hepatic impairment has not been characterized. Notably, Acoramidis is a time-dependent inhibitor of CYP2C9, and concomitant use with UGT inducers or strong CYP3A inducers should be avoided [33].

The ATTRibute-CM trial was a phase 3 double-blind, placebo-controlled study that randomized 632 patients with ATTR-CM in a 2:1 ratio to receive Acoramidis hydrochloride 800 mg twice daily or a matching placebo for 30 months [18]. All randomized participants were included in the intention-to-treat population; however, the primary analysis was performed with 611 patients in a 2:1 ratio, due to the exclusion of 21 patients with a baseline eGFR <30 mL/min/1.73 m^2^ [18]. The primary analysis used the stratified Finkelstein–Schoenfeld hierarchical method across four components: all-cause mortality, cardiovascular-related hospitalization, NT-proBNP level change from baseline, and 6 min walk distance [18]. Acoramidis was superior to placebo (*p* < 0.001), with a win ratio of 1.8 (95% CI, 1.4–2.2). 63.7% of pairwise comparisons favored Acoramidis, with only 35.9% favoring placebo [18]. Acoramidis significantly reduced the composite of all-cause mortality or first cardiovascular-related hospitalization compared with placebo (35.9% vs. 50.5%; HR, 0.64; 95% CI, 0.50–0.83; *p* = 0.0008), corresponding to a number needed to treat of 7 over 30 months [34]. Kaplan–Meier curves separated as early as month 3 and continued to diverge through month 30, driven by early reduction in cardiovascular-related hospitalizations (HR, 0.60; 95% CI, 0.45–0.80; *p* = 0.0005). At 30 months, all-cause mortality was numerically lower with Acoramidis than placebo, corresponding to survival rates of 80.7% versus 74.3% (HR, 0.77; 95% CI, 0.54–1.10; *p* = 0.154), although this difference did not reach statistical significance [8,18].

Following ATTRibute-CM, 389 participants enrolled in an open-label extension (OLE) study, with 263 continuing Acoramidis and 126 beginning Acoramidis from placebo [34]. Upon initiation of Acoramidis in the original placebo group, there was a prompt increase in serum transthyretin and stabilization of NT-proBNP levels, although quality of life (KCCQ-OS) remained below that of the continuous acoramidis group [34,35]. Long-term follow-up through 54 months further demonstrated that continuous Acoramidis treatment was associated with sustained reductions in all-cause mortality (HR, 0.55; 95% CI, 0.42–0.74; *p* < 0.001) and first cardiovascular-related hospitalization (HR, 0.53; 95% CI, 0.42–0.69; *p* < 0.001) [35]. A post hoc recurrent-event analysis revealed that Acoramidis reduced the cumulative burden of cardiovascular mortality or recurrent cardiovascular-related hospitalization by 49% through month 30 (HR, 0.51; 95% CI, 0.43–0.62; *p* < 0.0001), translating to 53 fewer cardiovascular events per 100 treated participants (95% CI, 29–79) [36]. This difference was already numerically apparent by month 1 and grew progressively over time, underscoring the importance of early diagnosis and prompt initiation of therapy [35,36].

Acoramidis was well tolerated in ATTRibute-CM, with a similar incidence of adverse events between Acoramidis and placebo (98.1% vs. 97.6%) and serious adverse events less frequent with Acoramidis than placebo (54.6% vs. 64.9%) [18]. Gastrointestinal adverse reactions were slightly more common with Acoramidis, including diarrhea (11.6% vs. 7.6%) and upper abdominal pain (5.5% vs. 1.4%). Most side effects were mild and resolved without discontinuation, and discontinuation rates due to adverse events were similar between groups (9.3% vs. 8.5%) [33]. An important laboratory finding was an increase in serum creatinine (mean 0.2 mg/dL) and decrease in eGFR (mean 8.2 mL/min/1.73 m^2^) within the first 28 days of therapy, which subsequently stabilized and is believed to reflect inhibition of tubular creatinine secretion rather than true nephrotoxicity [33]. No new safety signals were identified in the long-term OLE through month 42 [34].

Acoramidis carries an average wholesale price of approximately $225,000 annually [37]. For Medicare Part D beneficiaries, the Inflation Reduction Act’s $2000 annual out-of-pocket cap (effective 2025) has since substantially reduced this cost burden [38]. Otherwise, Acoramidis faces the same access barriers described for Tafamidis, with manufacturer copayment assistance unavailable to Medicare and Medicaid beneficiaries and independent charity foundations limited by income eligibility.

### 2.3. Diflunisal

Diflunisal is a nonsteroidal anti-inflammatory drug (NSAID) that has been repurposed as a TTR kinetic stabilizer, binding within the two thyroxine-binding sites of the tetramer to prevent dissociation and subsequent amyloid fibril formation [22]. Diflunisal is rapidly and completely absorbed after oral administration, reaching peak plasma concentrations within 2–3 h, and has a relatively long plasma half-life of 8–12 h [39]. Its pharmacokinetics are nonlinear and concentration-dependent, such that doubling the dose more than doubles drug accumulation, and several days are required to reach steady state [39]. Diflunisal is more than 99% protein-bound and is eliminated renally, predominantly as glucuronide conjugates (~90% of the dose). Renal impairment prolongs its half-life and results in metabolite accumulation, necessitating dose reduction [39].

In a phase 3 randomized, placebo-controlled trial of 130 patients with hereditary ATTR polyneuropathy, Diflunisal (250 mg twice daily) significantly reduced the rate of neurological progression compared with placebo over two years [40]. However, no randomized controlled trials have evaluated Diflunisal specifically in ATTR-CM, and research is limited to open-label, single-center studies and retrospective analyses. In an early observational study of 13 patients treated for one year, Diflunisal was well tolerated with minimal changes in ejection fraction, interventricular septal thickness, and left ventricular mass [41]. A larger retrospective cohort study of 104 wild-type ATTR-CM patients from Boston University, with 35 patients treated with Diflunisal, demonstrated improved survival after adjustment for baseline covariates (adjusted HR 0.18, 95% CI 0.06–0.51; *p* = 0.0006) and overall stability in echocardiographic markers, although 40% of patients eventually discontinued therapy [42]. A systematic review of six studies and 400 patients further supported potential efficacy, reporting associations with decreased mortality and stabilization of cardiac biomarkers, with no severe reactions [43]. A recent prospective pilot study employing serial cardiac magnetic resonance (CMR) imaging, cardiac biomarkers, and functional capacity assessments reported that Diflunisal was overall well tolerated and tended to stabilize or slow the progression of amyloid cardiac disease, as reflected by CMR parameters (left ventricular ejection fraction, extracellular volume, and T2 values). However, a statistically significant but clinically modest decline in renal function was observed [44].

Nonetheless, Diflunisal carries the inherent risks of NSAIDs, including renal toxicity, gastrointestinal bleeding, fluid retention, and thrombocytopenia, which are particularly relevant in a heart failure population [22]. The 2023 ACC Expert Consensus Decision Pathway acknowledges Diflunisal as a low-cost alternative (approximately $25–50 per month) for patients who cannot afford or access approved TTR stabilizers. However, the ACC Pathway recommends that it be avoided in those with impaired kidney function (eGFR <45 mL/min/1.73 m^2^), a history of gastric bleeding, recent heart failure decompensation, or high-dose diuretic use [30]. With the approval of Tafamidis, Acoramidis, and Vutrisiran, Diflunisal is generally reserved for selected patients unable to access these approved disease-modifying therapies [8,21].

### 2.4. Tolcapone

Tolcapone, a catechol-O-methyltransferase (COMT) inhibitor marketed as Tasmar for Parkinson’s disease, has been identified as a potent kinetic stabilizer of the TTR tetramer [45]. In vitro and ex vivo studies demonstrated that Tolcapone binds to TTR in human plasma, stabilizes both wild-type and the Val122Ile cardiomyopathy-associated variant, and reduces TTR-induced cytotoxicity. Unlike many stabilizers, it binds both T4-binding sites with similarly high affinity, without the typical negative cooperativity [45,46]. A phase IIa proof-of-concept study of 17 participants demonstrated significant TTR stabilization with no adverse events over a short observation period [47]. A distinguishing feature of Tolcapone is its ability to penetrate the blood–brain barrier, with the potential of stabilizing TTR produced by the choroid plexus, as leptomeningeal and ocular TTR production are not meaningfully suppressed by hepatically targeted gene-silencing therapies [10,48].

Despite these promising early findings, several limitations restrict Tolcapone’s broader clinical use. It has a short elimination half-life of approximately 2–3 h, requiring multiple daily doses [49]. Its use is also limited by a well-documented risk of idiosyncratic hepatotoxicity, including rare fatal fulminant liver failure, leading to an FDA black-box warning and a requirement for liver function monitoring every 2–4 weeks [50]. No long-term outcome trials or phase 3 studies have evaluated Tolcapone in ATTR-CM, and current guidelines, including the 2025 ACC Concise Clinical Guidance, do not include Tolcapone among recommended disease-modifying therapies [21].

### 2.5. Epigallocatechin-3-Gallate (Green Tea Extract)

Epigallocatechin-3-gallate (EGCG), the most abundant catechin in green tea, binds to TTR and inhibits TTR aggregation in vitro and in cell culture [51]. Unlike conventional stabilizers that bind within the thyroxine-binding channel, crystallographic analysis demonstrated that EGCG binds to distinct surface sites [52]. Preclinical work suggests it may also disaggregate pre-formed TTR amyloid fibrils [53,54]. Early clinical observations were limited to small, uncontrolled studies. An early observational study of 19 ATTR-CM patients consuming green tea/extract for 12 months showed no progression in LV wall thickness and a 12.5% decrease in LV myocardial mass in a subgroup [55]. A later study of 25 ATTRwt patients receiving 600 mg EGCG daily reported a 6% decrease in LV myocardial mass [56]. However, a single-center retrospective study of 30 ATTR-CM patients treated with EGCG found it was well-tolerated but not associated with improved survival [57]. Given the limited and observational evidence, variable oral bioavailability, lack of standardized dosing, and potential hepatotoxicity at higher doses [58], the 2023 ACC Expert Consensus Decision Pathway does not recommend EGCG as part of standard of care for ATTR-CM [30].

### 2.6. Curcumin

Curcumin, a polyphenolic compound derived from turmeric (Curcuma longa), binds to the thyroxine-binding site of TTR and inhibits denaturant-induced structural changes in the tetramer [59]. Beyond tetramer stabilization, curcumin has also been shown in vitro to suppress TTR amyloid fibril formation by redirecting aggregation toward small, non-toxic “off-pathway” oligomers and, like EGCG, to promote disaggregation of pre-formed TTR amyloid fibrils [53]. In aged ATTRv (formerly familial amyloid polyneuropathy) transgenic mouse models, curcumin reduced TTR aggregate deposition, avoided tissue toxicity, and remodeled amyloid material through enhanced macrophage-mediated clearance [60,61]. However, no clinical trials have evaluated curcumin specifically in ATTR-CM. Its poor oral bioavailability, rapid systemic metabolism, and limited tissue distribution remain major barriers to therapeutic development [5,62]. Nanoformulation strategies are being explored to improve its pharmacokinetic profile, but their clinical relevance in ATTR amyloidosis remains unproven [63]. Overall, while curcumin is unlikely to be clinically useful in its native form, its multi-target mechanistic profile may guide the development of optimized derivatives.

## 3. Gene Silencers (RNA-Based Therapies)

Gene silencing therapies represent a mechanistically distinct approach to ATTR-CM treatment. Rather than stabilizing the TTR tetramer, these agents reduce hepatic TTR protein production by targeting TTR mRNA for degradation within hepatocytes [64,65]. This results in a marked reduction (approximately 74–87%) in circulating TTR concentrations, thereby reducing the substrate available for amyloid fibril formation [64,65,66]. Two classes of RNA-based therapeutics are in clinical use: small interfering RNA (siRNA) molecules and antisense oligonucleotides (ASOs) [21].

### 3.1. Patisiran

Patisiran (Onpattro) is a double-stranded small interfering RNA (siRNA) encapsulated in a lipid nanoparticle (LNP) that targets the 3′ untranslated region of both mutant and wild-type TTR messenger RNA (mRNA) in hepatocytes. Through the RNA interference (RNAi) pathway, Patisiran promotes degradation of TTR mRNA and reduces hepatic production of circulating transthyretin protein [64,67]. In 2018, Patisiran became the first FDA-approved RNAi therapy for adults with ATTRv and polyneuropathy, after the phase 3 APOLLO trial demonstrated significant halting or reversal of neuropathy progression [64], with long-term benefit sustained in the open-label extension [68]. Although initially approved for polyneuropathy, a prespecified cardiac subpopulation of 126 APOLLO patients showed Patisiran was associated with reduced left ventricular wall thickness, improved global longitudinal strain, and lower NT-proBNP compared with placebo at 18 months [69].

Patisiran is administered intravenously at 0.3 mg/kg every three weeks in patients weighing <100 kg, with a fixed maximum dose of 30 mg in those weighing ≥100 kg. It is administered over 80 min and requires premedication with dexamethasone, acetaminophen, an H1-receptor antagonist, and an H2-receptor antagonist to reduce infusion-related reactions, which occurred in approximately 19% of patients. Patisiran demonstrates linear, dose-proportional pharmacokinetics over the range studied. Within its lipid nanoparticle formulation, Patisiran preferentially distributes to the liver. More than 95% of circulating Patisiran remains lipid-complex associated, while direct plasma protein binding is low (≤2.1%). Steady state is reached by approximately 24 weeks with ~3.2-fold accumulation. It has a mean terminal half-life of approximately 3.2 days, is metabolized primarily by nucleases, and undergoes minimal renal excretion (<1% unchanged in urine). Mild-to-moderate renal impairment and mild hepatic impairment do not require dose adjustment, and clinically significant CYP450-mediated drug interactions are not expected; however, severe renal and moderate-to-severe hepatic impairment have not been adequately studied. Because TTR transports retinol-binding protein, Patisiran lowers serum vitamin A levels, and supplementation with the recommended daily allowance of vitamin A is advised during treatment [70].

The subsequent APOLLO-B trial was a phase 3, double-blind study of 360 patients with variant or wild-type ATTR-CM, randomized 1:1 to intravenous Patisiran (0.3 mg/kg, maximum 30 mg) or placebo every 3 weeks for 12 months [66]. The trial met its primary endpoint, demonstrating a smaller decline in 6 min walk distance at 12 months with patisiran than with placebo, with a Hodges-Lehmann median difference of 14.69 m favoring patisiran (95% CI, 0.69–28.69, *p* = 0.02). Quality of life, assessed by the KCCQ-OS score, also favored Patisiran, with a least-squares mean change of 3.7 points (95% CI, 0.2–7.2; *p* = 0.04) [66]. However, significant benefits were not observed for the composite secondary endpoints involving mortality and cardiovascular events [66]. Adverse events were common in both groups (91% patisiran, 94% placebo) and generally mild to moderate, including muscle spasms, arthralgias, and infusion-related reactions [66]. Despite meeting its functional and quality-of-life endpoints in APOLLO-B, the FDA declined the approval of patisiran for ATTR-CM, due to concerns that the observed effect size on primary and secondary outcomes lacked clinical meaningfulness [8,71]. Alnylam subsequently announced it would not pursue an expanded cardiomyopathy indication for patisiran in the United States, although it maintains an important role in ATTRv-associated polyneuropathy.

### 3.2. Vutrisiran

Vutrisiran (Amvuttra) is a next-generation, subcutaneously administered siRNA therapy that suppresses hepatic production of both wild-type and variant transthyretin at the mRNA level. Its triantennary N-acetylgalactosamine (GalNAc) ligand promotes targeted uptake by hepatocytes through the asialoglycoprotein receptor, while chemical modifications enhance potency and allow administration once every three months [19]. Although initially approved in 2022 for ATTRv with polyneuropathy, Vutrisiran received approval for ATTR-CM in 2025, becoming the first RNA interference therapy for this indication [21,72].

The approval was based on the landmark HELIOS-B trial, a phase 3, double-blind, placebo-controlled study that randomized 655 patients with ATTR-CM (both ATTRwt and ATTRv) in a 1:1 ratio to Vutrisiran 25 mg or placebo every 12 weeks for up to 36 months [19]. The primary endpoint was a composite of all-cause mortality and recurrent cardiovascular events, tested in the overall population and the monotherapy population (patients not receiving Tafamidis at baseline). Vutrisiran significantly reduced this composite endpoint compared with placebo in both the overall population (HR 0.72; 95% CI, 0.56–0.93; *p* = 0.01) and the monotherapy population (HR 0.67; 95% CI, 0.49–0.93; *p* = 0.02) [19]. An updated HELIOS-B analysis using a later data cutoff (42-month follow-up for 96.3% of patients) further strengthened these findings: Vutrisiran reduced all-cause mortality by 36% (HR 0.64; 95% CI, 0.46–0.88), cardiovascular mortality by 33% (HR 0.67; 95% CI, 0.47–0.96), cardiovascular hospitalizations (RR 0.75; 95% CI, 0.62–0.91; *p* < 0.01), and heart failure hospitalizations (RR 0.67; 95% CI, 0.52–0.86), with generally consistent treatment effects regardless of baseline Tafamidis use [73]. Although HELIOS-B was not powered to evaluate combination therapy with Vutrisiran plus Tafamidis, the lack of statistically significant clinical endpoint improvement among patients receiving Vutrisiran on top of baseline Tafamidis suggests routine combination silencer–stabilizer therapy should not currently be recommended [21].

Vutrisiran is administered as a 25 mg subcutaneous injection once every three months. Following subcutaneous administration, Vutrisiran is rapidly absorbed, reaching peak plasma concentrations at a median of approximately 4 h, and preferentially distributes to the liver via GalNAc-mediated hepatocyte uptake. Plasma protein binding is concentration-dependent and approaches approximately 80% at lower concentrations. It has a median terminal half-life of approximately 5.2 h, with no plasma accumulation observed with quarterly dosing. Vutrisiran is metabolized by endo- and exonucleases into short nucleotide fragments, with approximately 19.4% excreted unchanged in the urine. Mild-to-moderate renal and hepatic impairment do not require dose adjustment, and clinically significant CYP450-mediated drug interactions are not expected; however, severe renal impairment, end-stage renal disease, severe hepatic impairment, and prior liver transplantation have not been studied. Because TTR transports retinol-binding protein, Vutrisiran reduces serum vitamin A levels by approximately 62–65% at steady state, and supplementation with the recommended daily allowance of vitamin A is advised during treatment [72].

Beyond the primary endpoint, Vutrisiran showed consistent benefit across secondary measures of survival, functional capacity, and health-related quality of life, with smaller declines in 6 min walk distance and KCCQ-OS score compared with placebo (treatment differences of 26.5 m and 5.8 points, respectively; both *p* < 0.001), including in the monotherapy population [19]. In a prespecified analysis at 30 months, a greater proportion of Vutrisiran-treated patients maintained or improved KCCQ-OS scores compared with placebo using both a 5-point threshold (63.5% vs. 46.6%; *p* < 0.001) and a 10-point threshold (74.6% vs. 60.7%; *p* < 0.01) [74]. Secondary echocardiographic analyses at 30 months demonstrated that Vutrisiran favorably affected cardiac structure and function, attenuating declines in left ventricular ejection fraction (least-squares mean difference, 2.0%; *p* = 0.02), global longitudinal strain (1.2%; *p* < 0.01), and stroke volume (4.1 mL; *p* < 0.01), while limiting increases in wall thickness (−0.4 mm; *p* = 0.03) and LV mass index (−10.6 g/m^2^; *p* < 0.01) [75,76]. Vutrisiran also favorably affected cardiac biomarkers. At 30 months, patients receiving Vutrisiran had lower relative increases in NT-proBNP and troponin I compared with placebo (geometric mean fold-change ratios of 0.68 for both; *p* < 0.0001) [77].

Regarding safety, the incidence of adverse events in the primary HELIOS-B analysis was similar between groups (99% Vutrisiran vs. 98% placebo) [19]. Most were mild to moderate, often consistent with the natural history of ATTR-CM, and included peripheral edema, dizziness, falls, and diarrhea [78]. A pooled analysis of 707 patients from HELIOS-A and HELIOS-B, with treatment durations up to 58 months and more than 1500 patient-years of exposure, found Vutrisiran was well tolerated with no new safety signals and infrequent, generally mild injection-site reactions [78]. A prespecified renal analysis also added an important consideration given the frequent coexistence of ATTR-CM and renal dysfunction. Vutrisiran reduced the proportion of patients experiencing a ≥40% decline in eGFR compared with placebo in both the overall population (12.7% vs. 21.2%; *p* = 0.0041) and the monotherapy population (12.0% vs. 21.9%; *p* = 0.0102). Among patients who developed CKD stage ≥4, Vutrisiran was associated with a significantly lower risk of the primary composite outcome (HR, 0.467; 95% CI, 0.258–0.845) [79].

### 3.3. Inotersen

Inotersen (Tegsedi) is a 2′-O-methoxyethyl-modified antisense oligonucleotide (ASO) that binds the 3′ untranslated region of TTR mRNA and promotes RNase H1-mediated degradation, reducing hepatic production of both mutant and wild-type transthyretin [10,65]. The NEURO-TTR trial, an international, double-blind, placebo-controlled phase 3 study of 172 patients, led to FDA approval in October 2018 for ATTRv-associated polyneuropathy. Inotersen significantly reduced neurologic progression and quality-of-life deterioration with sustained neurological benefit in the open-label extension [65,80]. The cardiac effects of Inotersen remain less well established. In a single-center open-label study of 33 patients with ATTR-CM, Inotersen was associated with an 8.4% mean reduction in LV mass and a 20.2 m increase in 6 min walk distance at 2 years; however, no randomized cardiovascular outcomes trial has been performed [65,81].

Inotersen is administered as a 284 mg subcutaneous injection once weekly. Following subcutaneous administration, it is rapidly absorbed, reaching peak plasma concentrations within approximately 2–4 h, with dose-proportional systemic exposure over the studied range. Inotersen is highly plasma protein-bound (>94%) and distributes broadly to tissues, with the highest concentrations observed in the kidney and liver. Steady state is reached after approximately 3 months, with no accumulation in plasma Cmax or AUC. It has a mean terminal elimination half-life of approximately 32.3 days and is metabolized primarily by nucleases into shorter nucleotide fragments, with <1% of the administered dose excreted unchanged in the urine within 24 h. Mild-to-moderate renal impairment and mild hepatic impairment do not require dose adjustment, and clinically significant CYP450-mediated drug interactions are not expected; however, severe renal impairment, end-stage renal disease, moderate-to-severe hepatic impairment, and prior liver transplantation have not been studied. Inotersen carries an FDA boxed warning for severe thrombocytopenia and glomerulonephritis and is available through a restricted REMS program requiring weekly platelet monitoring and renal function, urinalysis, and urine protein-creatinine ratio (UPCR) monitoring every two weeks; liver enzymes and bilirubin also require periodic monitoring. Because TTR transports retinol-binding protein, inotersen lowers serum vitamin A levels, and supplementation with the recommended daily allowance of vitamin A is advised during treatment [82].

The clinical utility of Inotersen must be weighed against important safety concerns. In NEURO-TTR, five deaths occurred in the Inotersen group compared with none in the placebo. One patient had a fatal intracranial hemorrhage associated with severe thrombocytopenia (platelet count <10,000/mm^3^), prompting implementation of frequent platelet monitoring [65]. Severe thrombocytopenia occurred in 3% of Inotersen-treated patients, and glomerulonephritis in another 3%, including one case that progressed to permanent hemodialysis; overall, 14% discontinued treatment due to adverse events [65]. Consequently, Inotersen is subject to a Risk Evaluation and Mitigation Strategy (REMS) requiring weekly platelet monitoring and regular renal assessment [22,30]. Its annual list price in the United States has been estimated at approximately $450,000, and treatment requires weekly self-injection with intensive laboratory monitoring, further limiting its practical appeal relative to newer agents such as Eplontersen and Vutrisiran [22,83].

### 3.4. Eplontersen

Eplontersen (Wainua) is a GalNAc-conjugated antisense oligonucleotide that targets TTR mRNA in hepatocytes, reducing production of both wild-type and variant transthyretin [84,85]. Compared with unconjugated ASOs, GalNAc conjugation enhances hepatocyte-specific delivery and increases potency approximately 20- to 30-fold, permitting a substantially lower dosing burden (45 mg subcutaneously every 4 weeks) relative to the first-generation ASO Inotersen (300 mg subcutaneously weekly) [84,86].

Eplontersen is administered as a 45 mg subcutaneous injection once monthly and is available as a single-dose autoinjector for patient or caregiver self-administration or as a prefilled syringe administered by a healthcare provider. Following subcutaneous administration, it is rapidly absorbed, reaching peak plasma concentrations at approximately 2 h, and distributes primarily to the liver and kidney cortex through GalNAc-mediated hepatocyte targeting. Eplontersen is highly plasma protein-bound (>98%), with slightly greater than dose-proportional increases in Cmax and AUC over the studied dose range and no accumulation with monthly dosing. It has a terminal elimination half-life of approximately 3 weeks and is metabolized by hepatic endo- and exonucleases into shorter oligonucleotide fragments, with <1% of the administered dose excreted unchanged in the urine within 24 h. Mild-to-moderate renal impairment and mild hepatic impairment do not require dose adjustment; however, severe renal impairment, end-stage renal disease, moderate-to-severe hepatic impairment, and prior liver transplantation have not been studied. Clinically significant transporter, protein binding, or CYP450-mediated drug interactions are not expected. In the NEURO-TTRansform trial, Eplontersen was not associated with severe thrombocytopenia or glomerulonephritis, and thrombocytopenia events were infrequent (2%), mild, and resolved without dose modification [65,84]. The most common adverse events were diarrhea, urinary tract infection, and nausea. Because TTR serves as a carrier for retinol-binding protein, Eplontersen-mediated TTR reduction also led to decreased serum vitamin A in 95% of patients, necessitating vitamin A supplementation [84,86].

Eplontersen was approved by the FDA in December 2023 for ATTRv with polyneuropathy, based on the phase 3 NEURO-TTRansform trial [85]. In this open-label study, 144 patients received Eplontersen and were compared with 60 patients who had received placebo in the earlier NEURO-TTR trial. At week 65–66, Eplontersen produced an 81.7% reduction in serum TTR and significantly reduced both neuropathy impairment and quality-of-life deterioration compared with the historical placebo group (mNIS+7 −24.8; 95% CI, −31.0 to −18.6; *p* < 0.001; Norfolk QoL-DN −19.7; 95% CI, −25.6 to −13.8; *p* < 0.001) [84]. In a post hoc cardiac subgroup analysis of NEURO-TTRansform, 65 weeks of Eplontersen was associated with improvements in LV ejection fraction (+4.3%; 95% CI, 1.40 to 21.01; *p* = 0.049) and stroke volume (+10.64 mL; 95% CI, 3.99 to 17.29; *p* = 0.002) relative to historical placebo, although these findings should be interpreted cautiously given the post hoc design, small sample, and use of a historical comparator [87,88].

These preliminary cardiac findings provided the rationale for the CARDIO-TTRansform trial, a pivotal phase 3, double-blind, placebo-controlled cardiovascular outcomes study of Eplontersen in ATTR-CM [88]. The trial enrolled 1432 participants with both ATTRwt and ATTRv cardiomyopathy, making it the largest ATTR-CM trial conducted to date; participants were randomized 1:1 to Eplontersen 45 mg or placebo subcutaneously every 4 weeks for up to 140 weeks. Notably, the trial permits unrestricted use of background TTR stabilizer, thereby allowing evaluation of the efficacy of combination gene-silencing and stabilizer therapy [88]. The primary composite endpoint is cardiovascular mortality and recurrent cardiovascular clinical events through 140 weeks, with secondary endpoints including 6 min walk distance, KCCQ overall summary score, all-cause mortality, and the primary endpoint in the stabilizer subgroup. Preliminary results from the CARDIO-TTRansform did not provide a statistically significant benefit on the composite outcome of CV mortality and recurrent CV events. This is the 1st largest ATTR-CM trial utilizing a silencer treatment, Eplontersen, in combination with TTR stabilizer, and proving combination treatment with a silencer and stabilizer treatment may not be beneficial. The full results of the trial will be released at the European Society of Cardiology (ESC) in August 2026 [88].

## 4. Gene Editing Therapy—Nexiguran Ziclumeran (Nex-z)

Nexiguran ziclumeran (Nex-z; formerly NTLA-2001), developed by Intellia Therapeutics, represents a potentially transformative approach as the first investigational in vivo CRISPR-Cas9 gene-editing therapy for ATTR-CM, designed to permanently suppress transthyretin production with a single intravenous treatment [89]. The therapy is delivered using a lipid nanoparticle containing messenger RNA encoding Streptococcus pyogenes Cas9 endonuclease and a single-guide RNA targeting the TTR gene in hepatocytes. This nonviral platform facilitates targeted hepatic delivery and rapid clearance and requires only minimal premedication, distinguishing it from viral vector–based gene therapies [89,90]. Preclinical data supported the durability of this strategy: in a nonhuman primate surrogate, a single dose achieved a maximum gene-editing rate of 73% in the liver and reduced circulating TTR by more than 94%, with effects stable through 12 months [89].

Nexiguran ziclumeran is administered as a single intravenous infusion. The phase 1 ATTR-CM expansion cohort received a fixed 55 mg dose, equivalent to approximately 0.7 mg/kg, infused over a minimum of 2 h. Patients received dexamethasone 8 mg or equivalent 8–24 h before treatment, followed by a glucocorticoid, H1-receptor antagonist, and H2-receptor antagonist approximately 1 h before infusion to reduce infusion-related reactions. Nex-z is delivered through a liver-tropic lipid nanoparticle that enables preferential hepatocyte uptake and is rapidly cleared following administration. Because TTR transports retinol-binding protein, Nex-z lowers vitamin A levels, and clinical trial protocols require supplementation with the recommended daily allowance of vitamin A [20,90].

Phase 1 clinical data in ATTR-CM demonstrated rapid, profound, and durable TTR reduction following a single 2 h infusion. In a phase 1 open-label trial, 36 patients with ATTR-CM received Nex-z and completed at least 12 months of follow-up [20]. Mean serum TTR levels decreased by 89% from baseline at day 28 (95% CI, −92 to −87) and by 90% at 12 months (95% CI, −93 to −87), with suppression maintained through 24 months, showing reductions that exceed those reported with currently approved silencing therapies (approximately 81% with Vutrisiran and 82% with Eplontersen) [20,21]. Cardiac biomarkers remained stable over 12 months, and 92% of patients demonstrated improvement or no change in NYHA functional class. Nex-z was generally well tolerated. Treatment-related adverse events included mild-to-moderate infusion-related reactions (14%) and transient elevations in hepatic transaminases (6%) that resolved without intervention [20]. Additional phase 1 data in ATTRv polyneuropathy further supported durability, with mean serum TTR decreased by 90% at day 28 and 92% through month 24, and clinically meaningful neurological improvement [90].

Despite these promising early results, subsequent phase 3 development has raised important safety concerns. The MAGNITUDE trial (NCT06128629) is a large, international, randomized, placebo-controlled phase 3 study initiated to determine whether Nex-z could reduce cardiovascular events and mortality in ATTR-CM [91,92]. However, in October 2025, enrollment and new infusions were paused after reports of severe hepatotoxicity in a small number of participants, including one death attributed to liver failure [91]. This safety signal highlights a central challenge of in vivo gene-editing therapies: the liver-directed delivery that enables highly efficient suppression of TTR production may also concentrate the risk of hepatic toxicity [92]. In addition, off-target editing remains a theoretical concern, although no evidence of off-target editing was detected in human primary hepatocytes exposed to concentrations up to three times the EC90 [89]. The future role of Nex-z will depend on whether ongoing investigations can demonstrate that these safety events are rare, predictable, and clinically manageable. The initial MAGNITUDE completion date is April 2028, with plans to follow patients for at least 15 years after treatment [20,91].

### Clinical-Stage CRISPR/Cas9-LNP Therapies Targeting TTR

Clinical-stage TTR-directed in vivo gene-editing programs remain limited but now include nexiguran ziclumeran, YOLT-201, and ART001. Each uses intravenous lipid nanoparticles to deliver an editor-encoding mRNA and a TTR-targeting guide RNA to hepatocytes, but the maturity and transparency of the available clinical evidence differ substantially [20,89,90,92,93,94,95,96]. The various CRISPR/Cas9-LNP therapies currently in clinical trial have been briefly compared by their editing payload and LNP delivery, the dose evaluated, the route and schedule of administration, and key details regarding each trial’s phase and status (Table 1).

## 5. Amyloid-Depleting Monoclonal Antibodies

Whereas TTR stabilizers and gene-silencing therapies primarily slow disease progression by reducing the formation of new amyloid fibrils, monoclonal antibodies represent a mechanistically distinct strategy aimed at promoting immune-mediated clearance of existing amyloid deposits [93,97]. This strategy is particularly intriguing in ATTR-CM, where established myocardial amyloid continues to contribute to ventricular stiffness and progressive dysfunction despite effective suppression of TTR production, and where diagnostic delays mean substantial amyloid burden is often present at diagnosis [98]. Amyloid-depleting antibodies have the potential to reduce myocardial amyloid burden, improve ventricular compliance, promote reverse remodeling, and address residual disease remaining after stabilization or silencing therapies [98,99].

### 5.1. Coramitug (PRX004/NNC6019

Coramitug (formerly PRX004/NNC6019) is a humanized monoclonal antibody originally developed by Prothena and later licensed to Novo Nordisk [100]. Unlike TTR stabilizers and gene-silencing therapies, Coramitug is designed to selectively bind misfolded TTR and deposited TTR amyloid fibrils while sparing native tetrameric TTR, thereby facilitating immune-mediated phagocytic clearance of existing deposits [93,98]. In a phase 1 open-label dose-escalation study of 21 patients with ATTRv, Coramitug was well tolerated across the evaluated dose range (0.1–30 mg/kg), with no maximum tolerated dose identified. Seven patients who received doses ≥ 3 mg/kg for at least 9 months demonstrated stabilization or improvement in global longitudinal strain and neuropathy impairment scores, providing preliminary evidence of biologic activity [93]. Subsequent evaluation in ATTR-CM was performed in a phase 2 double-blind, placebo-controlled trial that randomized 104 participants in a 1:1:1 ratio to Coramitug 10 mg/kg, 60 mg/kg, or placebo intravenously every four weeks for 52 weeks [98]. At the 60 mg/kg dose, Coramitug produced a significant 47% reduction in NT-proBNP relative to placebo (95% CI, −65% to −22%; *p* = 0.0017) and favorable echocardiographic changes; however, the co-primary endpoint of six-minute walk distance did not significantly improve, leaving uncertainty regarding the extent to which the biomarker and imaging benefits translate into meaningful functional outcomes [98]. Across phase 1 and phase 2 studies, Coramitug demonstrated an acceptable safety profile with adverse event rates generally comparable to placebo [93,98]. The phase 3 randomized CLEOPATTRA [100] has completed enrollment and is expected to conclude in 2027 [21,98,100].

Coramitug is administered intravenously every four weeks. In the phase 1 dose-escalation study, doses ranging from 0.1 to 30 mg/kg demonstrated approximately dose-proportional increases in systemic exposure, with a mean observed terminal half-life of approximately 31 days that was similar across dose levels. Pharmacokinetic/pharmacodynamic modeling indicated that doses ≥3 mg/kg achieved exposures predicted to occupy >90% of available amyloid targets, supporting sustained target engagement with monthly administration. Subsequent phase 2 evaluation used doses of 10 and 60 mg/kg intravenously every four weeks. Formal renal or hepatic dose-adjustment recommendations and CYP450 interaction profiles have not been established because Coramitug remains investigational [93].

### 5.2. NI006 (ALXN2220)

ALXN2220 (formerly NI006) is a recombinant human anti-ATTR monoclonal IgG1 antibody that selectively recognizes amyloid conformations of both wild-type and variant transthyretin while sparing physiologically folded TTR [97]. Originally identified through immune repertoire analyses of memory B cells from healthy older individuals, ALXN2220 was developed as an amyloid-depleting therapy designed to promote immune-mediated clearance of existing ATTR deposits, with preclinical studies demonstrating antibody-mediated phagocytosis of ATTR fibrils and depletion of tissue amyloid [97,101]. The first clinical evaluation was conducted in a phase 1 randomized, double-blind, placebo-controlled trial of 40 patients with wild-type or variant ATTR-CM and chronic heart failure [97]. Treatment was generally well tolerated, with no apparent drug-related serious adverse events and no antidrug antibodies; reported nonserious events included grade 1–2 cytokine release syndrome in three patients and dose-dependent musculoskeletal events. At doses ≥ 10 mg/kg every four weeks, ALXN2220 produced favorable changes in imaging biomarkers of cardiac amyloid burden, including reduced cardiac tracer uptake on bone scintigraphy and decreased extracellular volume on cardiac magnetic resonance imaging, while median NT-proBNP and troponin T declined over the 12-month study period [97]. Further support for this paradigm comes from observations of spontaneous antibody-associated regression of ATTR-CM, in which naturally occurring anti-ATTR antibodies were linked to substantial reversal of cardiac abnormalities [102,103]. To determine whether amyloid depletion translates into meaningful clinical benefit, ALXN2220 is being evaluated in the multinational, placebo-controlled phase 3 DepleTTR-CM trial, which uses a primary composite endpoint of all-cause mortality and cardiovascular clinical events [21,104].

In the phase 1 study, ALXN2220 was administered by intravenous infusion every four weeks. Doses ranging from 0.3 to 60 mg/kg demonstrated approximately dose-proportional increases in systemic exposure, as measured by Cmax and AUC. Following a single intravenous dose, serum concentrations declined in a biphasic manner, with a terminal elimination half-life ranging from approximately 15.5 to 19.2 days, consistent with the pharmacokinetic profile of a human IgG antibody. No antidrug antibodies were detected during the phase 1 study [97].

### 5.3. AT-02 (Pan-Amyloid Antibody)

AT-02, developed by Attralus, is a pan-amyloid removal peptide or an immunoglobulin fusion protein designed to bind amyloid deposits with high affinity and promote macrophage-mediated phagocytosis of amyloid fibrils [105,106]. The molecule incorporates the p5R peptide, binding both amyloid fibrils and amyloid-associated heparin sulfate proteoglycans through charge-dependent electrostatic interactions. These structural features are shared across amyloid deposits irrespective of the precursor protein. AT-02 has demonstrated broad reactivity against AL, ATTR, and AA amyloid in preclinical studies [105,107]. This pan-amyloid binding strategy may offer advantages over precursor-specific antibodies, particularly in patients with mixed amyloid burden or uncertain amyloid subtype [108]. AT-02 has completed a phase 1 clinical trial in patients with systemic amyloidosis, including individuals with ATTR-CM and AL-CM, with safety and exploratory efficacy assessed using serial cardiac magnetic resonance imaging with extracellular volume mapping [106,109]. If proof of concept is confirmed, pan-amyloid depletion could complement stabilizers and gene-silencing therapies by directly targeting the amyloid burden already present at diagnosis, addressing a significant unmet clinical need in patients presenting with advanced cardiac disease [15].

AT-02 (zamubafusp alfa) is administered by intravenous infusion. The phase 1 AT02-001 study evaluated the pharmacokinetics of escalating single doses in healthy volunteers and patients with systemic amyloidosis, followed by multiple-dose administration in patients with systemic amyloidosis. In the subsequent phase 2 open-label extension study, AT-02 is administered intravenously every two or four weeks. Detailed human pharmacokinetic parameters, including terminal elimination half-life, clearance, and dose proportionality, have not yet been reported in peer-reviewed clinical publications [110,111].

## 6. Patient Selection for Disease-Modifying Therapy

Selection of disease-modifying therapy should begin only after ATTR cardiac amyloidosis has been confirmed and light-chain amyloidosis has been excluded. Decisions should then consider disease stage, New York Heart Association functional class, ATTR genotype, cardiac and neurologic phenotype, renal and hepatic function, frailty, life expectancy, route and frequency of administration, adverse-effect profile, access, cost, and patient preference. No validated algorithm directly compares all available therapeutic classes or identifies the optimal treatment for an individual patient; therefore, these considerations constitute an evidence-informed framework rather than formal selection criteria [5,8,21,112].

Oral TTR stabilizers are generally appropriate for ambulatory patients with confirmed ATTRwt or ATTRv cardiomyopathy, particularly those with NYHA class I-III symptoms and preserved functional capacity. Tafamidis and acoramidis have demonstrated cardiovascular benefit in phase 3 trials, with the clearest benefit when therapy is initiated before advanced cardiac dysfunction develops [17,18]. Neither therapy removes established amyloid, and evidence remains limited in NYHA class IV disease, severe frailty, or very limited life expectancy. Diflunisal is an off-label alternative when approved stabilizers are inaccessible, but its nonsteroidal anti-inflammatory effects make it unsuitable for many patients with advanced heart failure, chronic kidney disease, gastrointestinal bleeding risk, or concomitant anticoagulation [40,41,42,43,44].

Vutrisiran may be considered for eligible patients with ATTRwt or ATTRv cardiomyopathy, including those with a mixed cardiac and polyneuropathic phenotype. Quarterly subcutaneous administration may be advantageous when infrequent dosing is preferred. Vitamin A supplementation is required, and evidence is limited in severe renal impairment, end-stage renal disease, severe hepatic impairment, and previous liver transplantation [19,72]. Whether Vutrisiran should replace a stabilizer or be added to one remains unresolved because HELIOS-B was not designed to determine the incremental benefit of combination treatment [19,21,73].

Patisiran, inotersen, and Eplontersen should not be considered established treatments for isolated ATTR-CM unless used within an approved indication or clinical trial. Their current approvals primarily address hereditary ATTR polyneuropathy. Inotersen additionally requires platelet and renal monitoring because of thrombocytopenia and glomerulonephritis risks. Gene-editing and amyloid-depleting therapies remain investigational and should presently be administered through clinical studies. CRISPR-based therapy may eventually suit carefully selected patients in whom the potential benefit of durable TTR suppression outweighs the uncertainty associated with irreversible editing and hepatic toxicity, whereas amyloid-depleting antibodies may be most relevant to patients with substantial residual myocardial amyloid burden [20,84,85,86,87,88,89,90,91,92,93,97,98,99,100,101,102,103,104,105,106,107,108,109,110,111]. All pharmacological therapeutics investigated for the treatment of ATTR-CM have been compared by their FDA status, their ideal candidates, strengths, and principal limitations (Table 2). 

## 7. Knowledge Gaps/Future Research Directions

The most pressing question in ATTR-CA is no longer whether the disease can be treated, but how the growing array of disease-modifying therapies should be optimally selected, combined, and sequenced [21,112]. Evidence for stabilizer–silencer combination therapy remains incomplete. HELIOS-B included patients receiving baseline Tafamidis, but the trial was not powered to determine the additive benefit of Vutrisiran plus Tafamidis [19,21]. CARDIO-TTRansform was designed to evaluate Eplontersen with unrestricted background stabilizer use and to provide a more direct assessment of this strategy through prespecified outcomes in the stabilizer subgroup [88].

Future studies should increasingly evaluate therapies targeting complementary stages of the amyloid cascade. TTR stabilizers and silencers limit formation of new amyloid fibrils, whereas amyloid-depleting agents such as ALXN2220, Coramitug, and AT-02 are designed to promote clearance of established deposits [97,98,105]. This creates a strong rationale for dedicated trials combining or sequencing production-suppressing therapy with amyloid depletion, particularly in patients with substantial residual myocardial amyloid. A related strategy would pair durable TTR suppression through CRISPR-based gene editing with subsequent amyloid depletion, although the hepatic safety concerns observed during nexiguran ziclumeran development require continued evaluation [20,92]. Sequential approaches, by first limiting further TTR production and subsequently targeting established deposits, represent another strategy that warrants prospective investigation. A more specialized combination may involve a blood–brain-barrier–penetrant stabilizer such as Tolcapone with a hepatically targeted silencer. Because Tolcapone can penetrate the blood–brain barrier and stabilize TTR, it could theoretically complement liver-directed silencing in compartments where locally produced TTR, particularly from the choroid plexus, is not meaningfully suppressed [10,45,48]. However, its hepatotoxicity and absence of long-term ATTR-CM outcome data currently limit its clinical applicability.

Beyond combination therapy, head-to-head comparisons of stabilizers and silencers remain necessary to establish optimal first-line treatment, while randomized evidence is also needed to define the role of adjunctive heart-failure therapies such as SGLT2 inhibitors in ATTR-CM [21,112]. Precision-medicine studies should further determine whether TTR genotype, phenotype, or biomarkers of disease progression can predict differential response to stabilization, silencing, gene editing, or amyloid depletion rather than assuming uniform benefit across a genetically heterogeneous disease [9,10]. Earlier diagnosis remains equally important, as delays in recognition postpone access to disease-modifying therapies, which appear most beneficial when initiated before substantial cardiac dysfunction develops [5,14]. AI-enhanced electrocardiographic and echocardiographic screening may help identify otherwise unrecognized ATTR-CA and shorten persistent diagnostic delays [14,113,114]. Future trials should also prioritize diverse enrollment and equitable access, particularly given the disproportionate burden of Val122Ile-associated ATTR-CA among African Americans [11,21].

## 8. Conclusions

Transthyretin cardiac amyloidosis has been transformed within a decade from an under-recognized, effectively untreatable cause of restrictive cardiomyopathy into one of the most therapeutically active areas of cardiovascular medicine. Advances in understanding of the amyloid cascade—including tetramer dissociation, monomer misfolding, and fibril deposition—have driven the development of multiple targeted therapeutic strategies that now span the disease pathway [8,23].

TTR stabilizers such as Tafamidis and Acoramidis have demonstrated clear reductions in mortality and cardiovascular hospitalizations, while gene-silencing therapies, including Vutrisiran and Eplontersen, suppress TTR production at its source, with Vutrisiran showing favorable clinical outcomes in HELIOS-B. Beyond these approaches, CRISPR-based gene editing with nexiguran ziclumeran offers the potential for durable single-dose TTR suppression, although recent hepatotoxicity signals highlight the importance of continued safety evaluation. At the same time, amyloid-depleting antibodies have introduced the possibility of actively removing established deposits, extending the prospect of disease modification beyond the prevention of further amyloid accumulation [17,18,19,97].

As a result, the central challenges facing the field are no longer whether ATTR-CM can be treated, but how emerging therapies should be optimally deployed. Head-to-head and combination studies, randomized evidence supporting adjunctive heart-failure management, validated treatment-response criteria, and improved access to diagnosis and care remain important unmet needs [21,112]. Continued progress will depend on earlier diagnosis through AI-enhanced screening, genotype- and biomarker-guided therapeutic selection, and the rational integration of complementary disease-modifying strategies. Equally important will be ensuring that these advances reach the populations most affected by ATTR-CM. Achieving these goals will require close collaboration among cardiologists, neurologists, geneticists, hematologists, and patients to translate the momentum of the past decade into durable and equitable improvements in clinical outcomes [15,21,113,114].

## Figures and Tables

**Figure 1:**
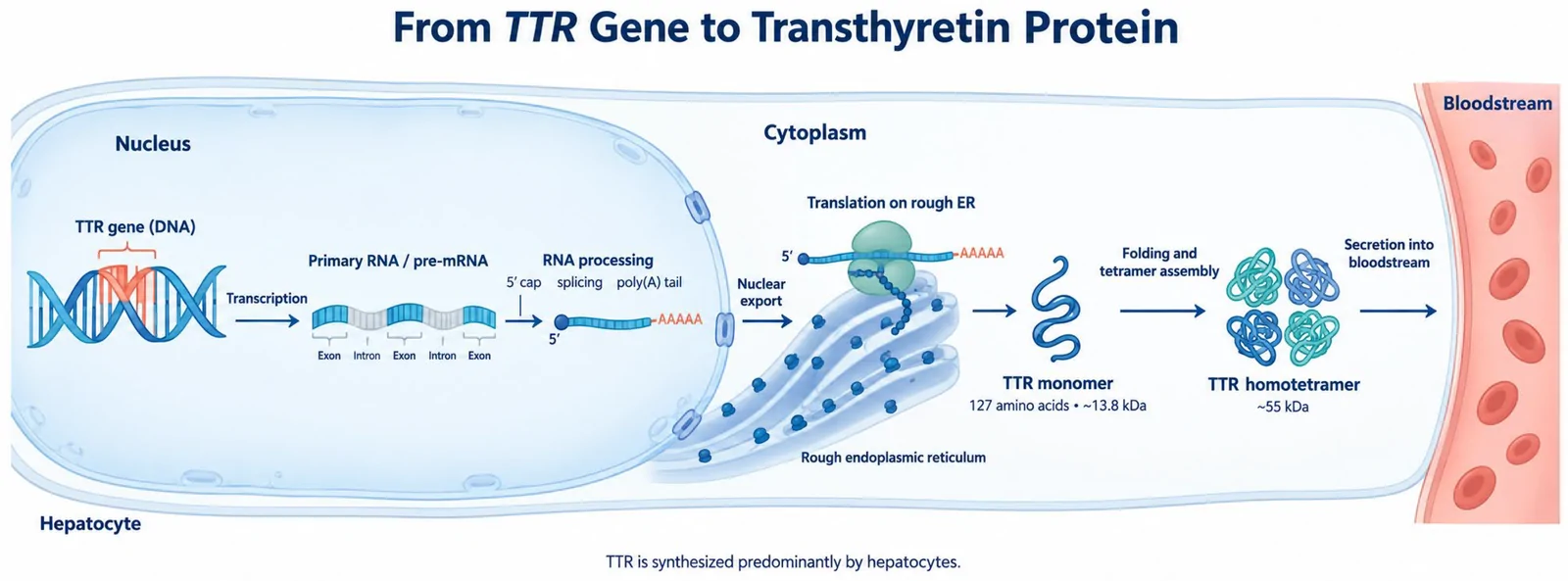
Hepatic biosynthesis and assembly of transthyretin. The *TTR* gene is transcribed in the hepatocyte nucleus to produce precursor messenger RNA, which undergoes 5′ capping, splicing, and polyadenylation. Mature *TTR* mRNA is exported to the cytoplasm and translated on the rough endoplasmic reticulum. Following processing and folding, four 127-amino-acid TTR monomers assemble into the approximately 55 kDa homotetramer, which is secreted into the circulation. The original illustration was created by the authors based on published descriptions of TTR synthesis and structure [7,8].

**Table 1:** Clinical-stage CRISPR/Cas9-LNP therapies targeting TTR.

Program and Trial	Editing Payload and LNP Delivery	Dose Evaluated	Route and Schedule	Phase, Status, and Key Details
Nexiguran ziclumeran (nex-z; NTLA-2001) NCT04601051; MAGNITUDE NCT06128629	SpCas9 mRNA plus TTR-targeting sgRNA in a liver-tropic ionizable LNP; hepatocyte uptake	Phase 1: 0.1, 0.3, 0.7, and 1.0 mg/kg; expansion and phase 3: 55 mg (approximately 0.7 mg/kg)	Intravenous infusion over at least 2 h; single dose; steroid plus H1/H2 premedication; daily vitamin A	Phase 1 and 3 in ATTR-PN and ATTR-CM; approximately 90% sustained TTR reduction. Phase 3 enrollment reinitiated in 2026 after a temporary hold; hepatic monitoring remains important [20,89,90,92,96].
YOLT-201 NCT06082050; NCT06539208	CRISPR/Cas editor mRNA plus TTR-targeting sgRNA in an ionizable LNP; ApoE/LDLR-mediated hepatocyte uptake	Numerical dose levels have not been publicly disclosed; dose escalation completed and an optimal biologically active dose was selected	Intravenous injection or infusion; single-dose escalation and expansion. Repeat dosing was reported in two low-dose IIT participants	Phase 1/IIa in ATTR-PN and ATTR-CM; greater than 90% TTR reduction in higher-dose cohorts reported by the sponsor; peer-reviewed efficacy data remain limited [94].
ART001 ChiCTR2400081216	SpCas9 mRNA plus TTR-targeting sgRNA in a proprietary LNP; hepatocyte TTR knockout	0.05–1.0 mg/kg across six ascending-dose cohorts	Intravenous administration; one dose per participant	Investigator-initiated study in 10 hereditary ATTR patients; mean TTR reductions of 84% and 92% at 0.7 and 1.0 mg/kg, respectively, at 72 weeks; small nonrandomized cohort [95].

**Table 2:** Comparison and potential patient-selection considerations for ATTR-CM therapies.

Therapy	Status for ATTR-CM	Potential Candidates	Strengths	Principal Limitations
Tafamidis	FDA approved	Confirmed ATTRwt or ATTRv; strongest evidence in ambulatory NYHA I-III disease	Oral once daily; longest outcome and clinical experience	High cost; delayed benefit; does not remove deposits; limited NYHA IV evidence
Acoramidis	FDA approved	Confirmed ATTRwt or ATTRv; ambulatory patients, especially earlier-stage disease	Oral; near-complete tetramer stabilization; phase 3 cardiovascular benefit	Twice-daily dosing; less real-world experience; does not remove deposits
Vutrisiran	FDA approved	ATTRwt or ATTRv; utilizated in mixed cardiac-neuropathic manifestations	Quarterly subcutaneous dosing; suppresses wild-type and variant TTR; outcome benefit	Vitamin A supplementation; limited severe renal/hepatic data; added value over stabilizer unknown
Diflunisal	Off label	Selected patients unable to access approved stabilizers and without major renal, gastrointestinal, or heart-failure contraindications	Inexpensive oral stabilizer	Fluid retention, renal injury, bleeding, and gastrointestinal toxicity; no definitive CM outcome trial
Patisiran	Not approved	Used for ATTRv polyneuropathy without cardiac involvement	Potent TTR reduction; supportive cardiac biomarker and imaging findings	Intravenous infusions and premedication; no ATTR-CM indication
Inotersen	Not approved	ATTRv polyneuropathy; limited role when cardiomyopathy predominates	Weekly self-administration	Thrombocytopenia and glomerulonephritis; intensive monitoring; no established CM outcome benefit
Eplontersen	Investigational	Used in ATTRv polyneuropathy without cardiac involvement; results pending (end of 2026) for ATTR-CM in CARDIO-TTRansform	Monthly dosing; substantial TTR suppression; background stabilizers permitted in CARDIO-TTRansform	ATTR-CM outcome benefit and incremental combination effect not established
Nexiguran ziclumeran	Investigational	Carefully selected clinical-trial participants	Potential one-time, durable TTR suppression	Irreversible editing; limited long-term data; hepatic safety concerns
Amyloid-depleting antibodies	Investigational	Patients with substantial established amyloid burden	Potential removal of existing deposits and reverse remodeling	Clinical efficacy unconfirmed; infusion burden; optimal sequencing unknown
EGCG/curcumin	Not approved	No established clinical role outside research	Accessible; mechanistic and preclinical signals	Uncertain formulation, bioavailability, dose, and clinical benefit

## Data Availability

No new data were created or analyzed in this study. Data sharing is not applicable to this article.
